# Thematic research clusters in very old populations (≥ 80 years): a bibliometric approach

**DOI:** 10.1186/s12877-021-02209-7

**Published:** 2021-04-21

**Authors:** Gregorio Gonzalez-Alcaide, Sergio Palacios-Fernandez, Jose-Manuel Ramos-Rincon

**Affiliations:** 1 Department of History of Science and Documentation, University of Valencia, Valencia, Spain; 2 Department of Internal Medicine, Hospital Universitari Sant Joan d’Alacant, Alicante, Spain; 3 Department of Internal Medicine, General University Hospital of Alicante-ISABIAL, Alicante, Spain; 4 Department of Clinical Medicine, Miguel Hernandez University of Elche, Alicante, Spain

**Keywords:** Elderly, Oldest old, Research output, Health research topics, Cardiovascular diseases, Cerebrovascular diseases, Dementias, Neoplasms, Geriatric syndromes, Psychosocial aspects of aging

## Abstract

**Background:**

Population aging will be one of humanity’s major challenges in the decades to come. In addition to focusing on the pathologies causing the greatest mortality and morbidity in this population, such as dementia, health research in elderly people must consider a myriad of other interlinked factors, such as geriatric syndromes, social aspects, and factors related to preserving quality of life and promoting healthy aging. This study aims to identify the main subject areas attracting research attention with regard to very old (≥ 80 years) populations.

**Methods:**

Documents assigned with the medical subject heading “Aged, 80 and over” were retrieved from MEDLINE and the Web of Science. This dataset was used to determine publication output by disease, geographic region, country, and discipline. A co-word analysis was undertaken to identify thematic research clusters.

**Results:**

Since the mid-2000s, there has been a boom in scientific output focusing specifically on very old populations, especially in Europe (43.7% of the documents) but also in North America (30.5%) and Asia (26%); other regions made only nominal contributions (0.5 to 4.4%). The USA produced the most research, while the most growth over the study period occurred in Japan, Spain, and China. Four broad thematic clusters were identified: a) geriatric diseases, health services for the aged, and social and psychological issues of aging; b) cardiovascular diseases; c) neoplasms, and d) bacterial infections & anti-bacterial agents.

**Conclusions:**

Scientific research in very old populations covers a wide variety of interrelated topics. In quantitative terms, the top subject areas have to do with cardiovascular and cerebrovascular diseases (including aortic valve stenosis and stroke), dementia, and neoplasms. However, other degenerative pathologies, geriatric syndromes, and different social and psychosocial aspects also attract considerable interest. It is necessary to promote more equal participation in global research on pathologies and topics related to very elderly populations, as the highest rates of population aging and the largest numbers of elderly people in the next decades will be in low- and middle-income countries.

**Supplementary Information:**

The online version contains supplementary material available at 10.1186/s12877-021-02209-7.

## Background

The World Health Organization (WHO) estimates that the proportion of inhabitants aged over 60 years will double between 2000 and 2050, from 11 to 22% of the world population (roughly 2 billion people). The most significant rise, moreover, will be in the most elderly people, with the proportion of octogenarians quadrupling in the same period of time, reaching a figure of 395 million people. About 80% of older people will be in low- and middle-income countries, which will experience the highest rates of population aging over the coming decades [1].

Apart from the main causes of death (cardiovascular diseases, cancers, respiratory diseases and dementias) [2–4], older people’s quality of life can be greatly impaired by degenerative pathologies associated with aging, posing important challenges to the health system [5]. Specific conditions include hearing and vision loss, neck and back pain, and osteoarthritis, among others. This population group may also present geriatric syndromes that do not fall neatly into specific disease categories, like frailty, sarcopenia, urinary incontinence, polypharmacy, falls, delirium, or pressure ulcers. Furthermore, as people’s age advances, so does their risk of increased mortality associated with accidents and falls and having several comorbidities and concurrent syndromes at once.

Different bibliometric studies have taken “aging” as the topic of reference to analyze scientific literature, which has considered this variable in association with “healthy aging,” a concept describing optimal physical, mental and social well-being [6–10]. Some papers have analyzed the evolution of scientific output, the contributions by country, and the impact of publications, taking as references the journals specializing in geriatrics and gerontology [11–14]. Others have studied specific diseases that are generally associated with the elderly population, especially dementias like Alzheimer’s disease [15–17], neuromuscular manifestations like sarcopenia [18], fractures and falls [19], and eye diseases [20], among others. Only Lund and Wang [21] have taken a comprehensive approach to analyzing scientific literature linked to the oldest population bracket (≥ 85 years), tracking the evolution of scientific output based on documents indexed in the Web of Science and identifying the most productive authors and journals along with the most highly cited papers.

In very old populations, it is essential to consider these medical aspects hand in hand with social factors, including people’s family and living environment along with their overall well-being [22, 23]. However, the traditional medical model remains disease-centered, which is insufficient for capturing the complexity of health in older people. The progressive aging of the population requires increasingly greater efforts to better understand and address age-related health problems, including through more multidisciplinary research that specifically addresses the health of this population group. Although a large body of bibliometric literature exists on specific diseases associated with older people [15, 24–27], few studies have attempted to provide a comprehensive vision of the area. Moreover, those that exist are often limited to publications in the area of Geriatrics and Gerontology [6, 7, 11–13, 21].

The overarching aim of this study is to characterize international scientific research in very old people over the last two decades, identifying the main topics addressed in research and the geographic distribution of the resulting publications.

## Methods

The methods applied in the study followed the steps described below.

### Identification of the dataset (documents) under study

**Fig. 1:**
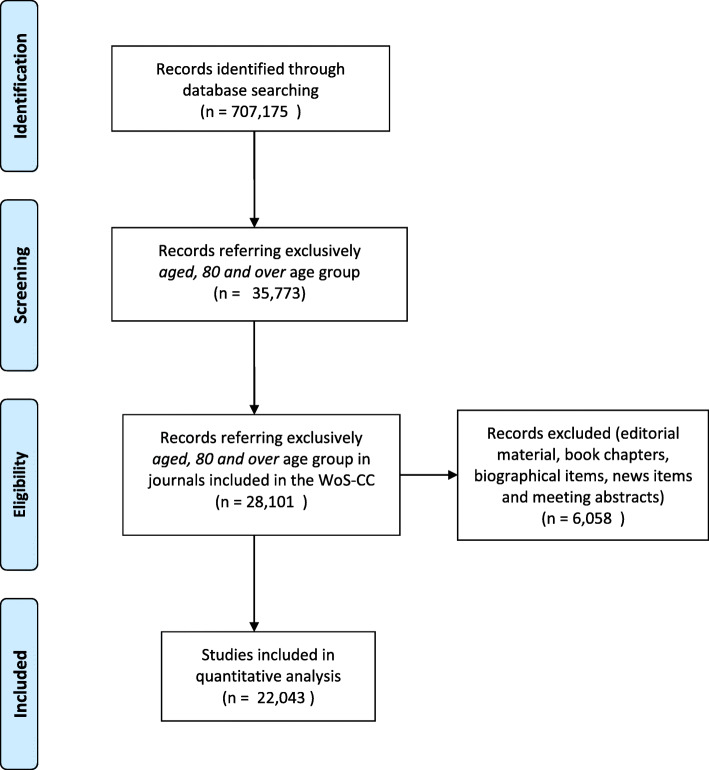
Flow chart for selection of study dataset

We used the National Library of Medicine’s MeSH thesaurus to identify documents (articles, reviews, and letters) that had been assigned the descriptor “Aged, 80 and over” (“a person 80 years of age and older”) between 2000 and 2019. The results comprised two groups of documents:

- Documents indexed in the MEDLINE database. The indicators obtained from this dataset enabled a general vision of the scientific output and subject areas in the biomedical literature with regard to this age group.
- Next, we generated a new dataset, excluding the retrieved documents that had also been assigned to other age groups (aged; middle aged; young adult; child; child, preschool; infant; infant, newborn) in order to specifically analyze the literature focusing exclusively on very old people, as a high number of publications jointly consider very old populations along with people from other age groups. Thus, epidemiological studies, basic research and other documents in which advanced age was not an exclusive key variable were excluded. To calculate the indicators, we opted to include only the documents in the Web of Science-Core Collection (WoS-CC). Although this database does not encompass all of the documents indexed in MEDLINE, it does offer some additional functionalities that are relevant to the study aims. For example, it classifies documents into areas of knowledge or disciplines, based on the subject area focus of the journals in which they were published. The WoS-CC also records all authors’ institutional affiliations and their corresponding countries, along with the number of citations that the documents receive. Figure 1 shows a flow chart for the selection of the study dataset.

Searches were undertaken using the Web of Science (Clarivate Analytics), which includes the MEDLINE database, on 24 June 2020.

### Analysis and treatment of bibliographic data

All bibliographic data were downloaded in order to review and standardize them prior to quantitative analysis. Multiple entries in some fields (institutional affiliations and MeSH descriptors) were individualized, and information on authors’ countries was extracted based on institutional affiliations, unifying all documents assigned to Scotland, England, Wales, and Northern Ireland as originating in the UK.

The WoS-CC databases include subject categories for both “Geriatrics & Gerontology” (in the case of the Science Citation Index Expanded (SCIE), which “includes the clinical, biochemical, histological, and psychological aspects of aging”) and “Gerontology,” linked to the Social Sciences Citation Index (SSCI) and covering “resources that are concerned with the sociological and psychological issues of aging.” For journals assigned to both categories, and generally for all journals assigned to more than one subject category, we made fractional assignments for the documents, proportional to the number of subject categories pertaining to the journal of publication.

### Indicators and analysis

#### Bibliometric analysis of scientific output

We analyzed the number of documents published per year and the frequency of the MeSH terms assigned to them, both for all documents retrieved and for the subset limited to the ≥80-year age group. With regard to the documents in the WoS-CC focusing exclusively on the very old population, we categorized them by geographic region (Africa, Asia, Europe, Latin America and the Caribbean, North America, and Oceania), country (considering the total number of publications and the number of publications per million population, according the World Bank Open Data, 2019), and thematic category of the publishing journals.

#### Characterization of subject areas: research clusters

To specifically identify the main subject areas attracting the most research attention, beyond the broader disciplines in which the documents were published, we carried out a co-word analysis to determine the frequency and co-occurrence of the MeSH terms assigned to them. First, a matrix was generated to quantify the joint appearances of 11,285 MeSH terms, and then a co-occurrence network was generated to show the relationships between high-frequency MeSH terms (> 30 documents). Next, to focus the analysis on the most relevant MeSH terms, we excluded from the analysis the generic descriptors referring to sex (“female” and “male”), epidemiological aspects, countries, diagnostic procedures and techniques, study designs, and statistical methods. Finally, Persson’s Party Clustering algorithm [28] was applied to identify research clusters based on the existing topic groups; these were labeled with concept terms that broadly described the aspects addressed. When interpreting the results, readers should keep in mind that a single document may be multi-assigned to different research clusters if it has been assigned MeSH terms for different diseases. Moreover, some diseases are not included in any of the clusters even though they are represented by a certain volume of documents in the literature, as they may not be linked to others or have a specific set of associated terms. In these cases, we have noted their presence narratively in the text.

### Ethics

As this was an analysis of available published research, no ethics approval was required. No authors were contacted for further information regarding their publications.

## Results

### Scientific output

We identified 707,175 documents that had been assigned the “Aged, 80 and over” descriptor in MEDLINE and 22,043 documents referring exclusively to this age group and published in journals included in the WoS-CC. The dataset included 16,383 articles (74.3%), 4456 letters (20.2%), 843 reviews (3.8%), and 361 proceedings papers (1.6%).

**Fig. 2:**
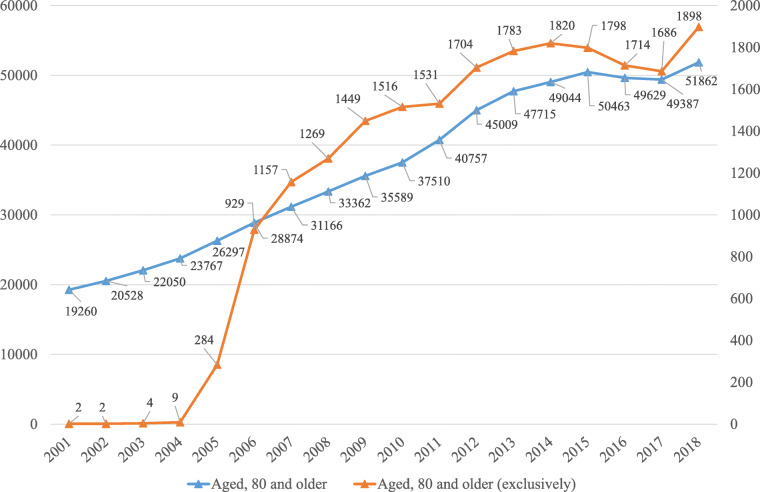
Annual evolution in the number of documents indexed in the WoS-CC and assigned the descriptor, “Aged, 80 and over”

The annual evolution in the number of documents published (Fig. 2) shows that while this age group has always been present in biomedical research, the boom in papers focusing exclusively on this population began in the mid-2000s.

The distribution of documents by geographic region (Table 1) shows that European researchers are responsible for the greatest total volume of scientific output (43.7% of the documents), followed by North America (30.5%) and Asia (26%). That said, Asian research output grew notably over the research period in both relative and absolute terms, while a certain decline is apparent in the other two regions. The rest of the regions studied made only nominal contributions (0.5 to 4.4%).

**Table 1:** Scientific research output focusing exclusively on people aged 80 and over, by geographic region

Geographic area	2000–2004		2005–2009		2010–2014		2015–2019		TOTAL	
	N	%	N	%	N	%	N	%	N	%
North America	7	46.7	1772	35.5	2484	30	2423	28.3	6686	30.5
Europe	4	26.7	2157	43.2	3805	46	3599	42.1	9565	43.7
Asia	4	26.7	1060	21.2	1947	23.5	2688	31.4	5699	26
Oceania	1	6.7	206	4.1	361	4.4	403	4.7	971	4.4
Africa	0	0	15	0.3	38	0.5	50	0.6	103	0.5
L. America & Caribbean	1	6.7	73	1.7	197	2.	236	2.8	507	2.3
TOTAL	15	0.1	4995	22.9	8268	37.9	8555	39.2	21,833	100

With regard to research participation by country (Table 2), the USA ranks first (27.7% of the documents), followed by Japan (14.1%) and different European countries (UK, Italy, Germany, France, and Spain at 5.9 to 7.9%). Japan, Spain, and China are the countries that have seen the greatest growth in scientific activity in this area, while Australia, Japan, and European countries stand out in terms of the number of publications per million population.

**Table 2:** Top 30 most productive countries for research output related to people aged 80 and over

Country	2000–2004		2005–2009		2010–2014		2015–2019		TOTAL		
	N	%	N	%	N	%	N	%	N	%	N publications per million inhabitants
USA	6	40	1627	32.	2233	27	2177	25.4	6043	27.7	18.4
Japan	2	13.3	594	11.9	1011	12.2	1471	17.2	3078	14.1	24.4
UK	2	13.3	526	10.5	647	7.8	558	6.5	1733	7.9	25.9
Italy	–	–	361	7.2	605	7.3	630	7.4	1596	7.3	26.5
Germany	–	–	239	4.8	524	6.3	563	6.6	1326	6.1	15.9
France	–	–	225	4.5	555	6.7	527	6.2	1307	6	19.5
Spain	1	6.7	193	3.9	508	6.1	587	6.9	1289	5.9	27.4
Australia	1	6.7	185	3.7	314	3.8	360	4.2	860	3.9	33.9
Canada	1	6.7	175	3.5	303	3.7	328	3.8	807	3.7	21.5
China	–	–	82	1.6	247	3	453	5.3	782	3.6	0.6
Netherlands	–	–	145	2.9	243	2.9	216	2.5	604	2.8	34.8
Switzerland	–	–	93	1.9	170	2.1	180	2.1	443	2	79.5
Sweden	–	–	97	1.9	160	1.9	184	2.1	441	2	42.9
Taiwan	1	6.7	115	2.3	164	2	161	1.9	441	2	19.1
Belgium	–	–	75	1.5	163	2	127	1.5	365	1.7	31.8
Turkey	1	6.7	78	1.6	154	1.9	131	1.5	364	1.7	4.4
South Korea	–	–	47	0.9	127	1.5	174	2	348	1.6	6.7
Greece	–	–	109	2.2	119	1.4	92	1.9	320	1.5	29.9
Israel	–	–	69	1.4	98	1.2	125	1.5	292	1.3	32.2
Brazil	–	–	37	0.7	125	1.5	130	1.5	292	1.3	1.4
Austria	–	–	51	1	79	1	92	1.1	222	1	25.0
Finland	–	–	36	0.7	83	1	88	1.	207	0.9	37.5
Portugal	–	–	17	0.3	59	0.7	110	1.3	186	0.8	18.11
Denmark	–	–	44	0.9	59	0.7	76	0.9	179	0.8	30.8
Singapore	–	–	26	0.5	53	0.6	83	1	162	0.7	28.4
Poland	–	–	15	0.3	60	0.7	65	0.8	140	0.6	3.7
Norway	–	–	25	0.5	52	0.6	62	0.7	139	0.6	26.0
New Zealand	–	–	21	0.4	60	0.7	57	0.7	138	0.6	28.1
Ireland	1	6.7	32	0.6	44	0.5	45	0.5	122	0.6	24.7
India	–	–	27	0.5	46	0.6	45	0.5	118	0.5	0.1

### Disciplines and topic areas

The analysis of disciplines in which the documents were published (Table 3) reveals the considerable dispersion of research interests, with investigators in 25 specialties participating in at least 1% of the documents. In absolute terms, the top categories are Cardiac & Cardiovascular Systems, Medicine, General & Internal, and Surgery. According to the size of the category (n journals), the most prominent topics, apart from Geriatrics & Gerontology and Gerontology, were Dermatology and Cardiac & Cardiovascular Systems, and among the moderately productive journals, Anesthesiology and Emergency Medicine.

**Table 3:** Main biomedical disciplines in which the documents about people aged 80 and over were published, according to WoS category for journal classification

Web of Science Category	N docs *	%	N Journals	Docs/Journal
Cardiac & Cardiovascular Systems	2395.7	10.9	138	17.4
Medicine, General & Internal	1924.1	8.7	165	11.7
Surgery	1824.4	8.3	210	8.7
Geriatrics & Gerontology †	1664.5	7.5	51	32.6
Dermatology	1410.8	6.4	68	20.7
Clinical Neurology	861	3.9	204	4.2
Gerontology †	812.8	3.7	36	22.6
Ophthalmology	742.8	3.4	60	12.4
Gastroenterology & Hepatology	641.7	2.9	88	7.3
Pathology	549.6	2.5	78	7
Oncology	505.4	2.3	244	2.1
Peripheral Vascular Disease	500.6	2.3	65	7.7
Radiology, Nuclear Medicine & Medical Imaging	498.9	2.3	133	3.7
Neurosciences	484	2.2	271	1.8
Orthopedics	450.6	2	82	5.5
Pharmacology & Pharmacy	410	1.9	270	1.5
Urology & Nephrology	409.3	1.9	85	4.8
Anesthesiology	355.9	1.6	32	11.1
Emergency Medicine	330.7	1.5	31	10.7
Hematology	327	1.5	76	4.3
Respiratory System	293.7	1.3	64	4.6
Infectious Diseases	259	1.2	93	2.8
Dentistry, Oral Surgery & Medicine	252.5	1.1	91	2.8
Nursing	250.1	1.1	123	2
Psychiatry	227.5	1	155	1.5

* Documents published in journals included in more than one WoS category were assigned a fractional value, in proportion to the number of categories pertaining to the journal. † These two categories are presented separately: Geriatrics & Gerontology (SCIE) and Gerontology (SSCI)

The co-word analysis of MeSH descriptors showed four prominent clusters, that bring together 77.5% of the documents, reflecting the main topic areas attracting the most research interest in relation to very old populations.

### Geriatric diseases, health services for the aged and social and psychological issues of aging

There is a heterogeneous cluster covering closely interrelated disorders and diseases that have a particularly strong impact on people of very advanced age (cluster A). The concepts are linked to diagnosis and treatment of the diseases as well as social and psychological aspects related to aging. The following topics stand out:

- Neurocognitive disorders (including dementias and especially Alzheimer’s disease), cognitive dysfunction, and depression all have a prominent presence in this cluster, as do descriptors referring to different tools for diagnosing dysfunctions, brain damage, and central nervous system disorders, and for assessing the severity of mental illnesses and measuring pain.
- Another area of research interest is fractures (particularly femoral fractures) caused by accidental falls. Papers focus on the procedures for their treatment, including orthopedic surgery using bone fixation devices to maintain proper fracture alignment.
- Numerous descriptors refer to the study of nutritional physiological phenomena associated with nutrient absorption and the assessment of nutritional status.
- The loss of skeletal muscle mass, especially in relation to sarcopenia, represents another specific area of research identified.
- Other descriptors have to do with the study of genetic factors associated with longevity, genetic predisposition, and conditions that can trigger the activation of disease.
- Finally, another thematic area concerns health services for the aged in the hospital setting as well as in institutional care and home care and social and psychological issues of aging.

### Cardiovascular diseases

Another cluster with a high density of interlinking terminology revolves around heart diseases and the surgical procedures used to address them (cluster B).

- Heart diseases. Different diseases and pathologies associated with heart valves and conditions linked to “myocardial ischemia” stand out among this cluster.
- Vascular diseases. This cluster also features different vascular diseases, like “aortic aneurysm,” both abdominal and thoracic; “iliac aneurysm”; and “carotid stenosis.”
- Cerebrovascular disorders. Finally, cerebrovascular disorders, especially “stroke” and “brain ischemia,” comprise a prominent area of study with its own specific lines of research.

### Neoplasms

A large set of terms linked to neoplasms, tumors, and carcinomas make up another cluster (cluster C), in which aspects such as pharmacological and surgical treatments, biomarkers, and antineoplastic agents are considered. This cluster also shows a specific line of research into “anti- vascular endothelial growth factor A” as a strategy to limit the angiogenesis associated with the use of drugs like bevacizumab, for treating both different cancers and age-related macular degeneration.

### Bacterial infections & anti-bacterial agents

Management of infections associated with “staphylococcal infections,” “prosthesis-related infections,” “urinary tract infections,” “klebsiella infections,” “gram-positive bacterial infections,” and “endocarditis, bacterial” are the main research topics in this cluster (cluster D), which covers topics like the combination of drugs, the sensitivity of microbial tests, and bacterial resistance.

Table 4 presents the scientific output and citation degree linked to all of the subject areas described. Overall, geriatric diseases and particularly Health services for the aged & social and psychological issues of aging and Neurocognitive & depressive disorders share the research spotlight with Cardiovascular diseases and Neoplasms.

**Table 4:** Scientific output of thematic clusters related to the descriptor “Aged, 80 and over”

Research clusters and sub-topics	N docs	%	N Citations	Citations per doc
A) Geriatric diseases, health services for the aged and social and psychological issues of aging	8381	38	143,734	17.1
- Health services for the aged & social and psychological issues of aging	5414	24.6	95,003	17.5
- Neurocognitive & depressive disorders	3094	14	62,695	20.3
- Muscle atrophy problems	874	4	16,071	18.4
- Falls & fractures	841	3.8	12,615	15
- Genetic factors	555	2.5	13,806	24.9
- Nutrition	349	1.6	5829	16.6
B) Cardiovascular diseases	6963	31.6	69,513	10
- Vascular diseases	5681	25.8	55,022	9.7
- Heart diseases	5380	24.4	56,313	10.5
C) Neoplasms	5205	23.6	34,882	6.7
D) Bacterial Infections & Anti-Bacterial Agents	1216	5.5	11,220	9.2

Additional file 1 presents the list of MeSH terms for diseases assigned to at least 50 of the documents on people aged 80 and over, with indicators for overall output and the number of documents that focus on the topic specifically for this age group. In general, the main diseases either fall under one of the clusters described above or are closely related to them. For example, they may deal with different neoplasms like “breast neoplasms” or “pancreatic neoplasms,” fractures like “spinal fractures,” or neurodegenerative diseases like “Parkinson’s disease.” There are also several diseases that are independently relevant, like “intestinal obstruction,” “deglutition disorders,” and “pulmonary embolism.”

### Diseases with greater incidence in people under 80 years of age and with sex-related differences

Some descriptors for age-related pathologies are more likely to be associated with descriptors like “aged” (“a person 65 through 79 years of age”) than exclusively “aged, 80 and over,” so they do not appear in the clusters described above. This is the case of “diabetes mellitus,” “osteoporosis,” “pulmonary disease, chronic obstructive,” “depression,” and “Parkinson’s disease.” Some significant differences between men and women have also been observed for certain diseases (Table 5). For example, research into some diseases is more frequently associated with women, such as heart valve diseases (e.g. “aortic valve stenosis”), degenerative diseases of the central nervous system (“dementia” and “Alzheimer’s disease”), falls and fractures (“accidental falls,” “hip fractures”), cardiovascular diseases (“hypertension”), neoplasms (“breast neoplasms”), and other deposition diseases (“calcinosis”). Other diseases are more researched in men, including some neoplasms (“skin neoplasms,” “adenocarcinoma,” “lung neoplasms,” “melanoma,” “prostatic neoplasms,” and “liver neoplasms”) and cardiovascular diseases (“aortic aneurysm abdominal” or “coronary artery disease”).

**Table 5:** Top 30 diseases studied in documents assigned with the descriptor “aged, 80 and over,” with differences according to sex

MeSH descriptor	N docs total	N docs only female	N docs only male	% only female*	% only male*	Dif % female – male^**†**^	***P*** value
Aortic Valve Stenosis	1134	260	191	22.9	16.8	6.1	< 0.001^‡^
Skin Neoplasms	951	396	499	41.6	52.5	−10.8	< 0.001^‡^
Dementia	697	91	48	13.1	6.9	6.2	< 0.001^‡^
Alzheimer Disease	575	103	65	17.9	11.3	6.6	0.001^‡^
Stroke	500	121	99	24.2	19.8	4.4	0.093
Carcinoma, Squamous Cell	435	196	189	45.1	43.4	1.6	0.63
Adenocarcinoma	417	165	214	39.6	51.3	−11.7	< 0.001^‡^
Accidental Falls	376	116	45	30.8	12	18.9	< 0.001^‡^
Cognition Disorders	365	35	35	9.6	9.6	0	1
Lung Neoplasms	365	117	171	32	46.8	−14.8	< 0.001^‡^
Heart Failure	352	89	89	25.3	25.3	0	1
Hypertension	325	85	60	26.1	18.5	7.7	0.018^‡^
Hip Fractures	323	63	17	19.5	5.3	14.2	< 0.001^‡^
Myocardial Infarction	295	80	78	27.1	26.4	0.7	0.86
Aortic Aneurysm, Abdominal	270	37	178	13.7	65.9	−52.2	< 0.001^‡^
Prosthesis Failure	264	114	103	43.2	39	4.2	0.33
Melanoma	253	100	134	39.5	53	−13.4	0.003^‡^
Hemorrhage	221	81	70	36.6	31.7	4.9	0.27
Depression	218	28	16	12.8	7.3	5.5	0.056
Coronary Artery Disease	215	35	58	16.3	27	−10.7	0.007^‡^
Cardiovascular Diseases	199	17	9	8.5	4.5	4	0.11
Breast Neoplasms	198	192	2	97	1	96	< 0.001^‡^
Carcinoma. Basal Cell	197	80	99	40.6	50.2	−9.6	0.054
Stomach Neoplasms	195	69	82	35.4	42	−6.	0.18
Neoplasm Recurrence, Local	192	96	80	50	41.7	8.3	0.10
Prostatic Neoplasms	168	–	164	0	97.6	−97.6	< 0.001^‡^
Liver Neoplasms	167	65	92	38.9	55.1	−16.8	0.003^‡^
Calcinosis	163	78	49	47.8	30.1	17.7	0.001^‡^
Cognitive Dysfunction	163	12	6	7.4	3.7	3.7	0.14
Neoplasm Invasiveness	162	72	68	44.4	42	2.5	0.65

* Percentage of male or female by MeSH descriptor, ^**†**^Percentage point differences between female and male, ^‡^ Statistically significant differences (*p* < 0.05)

## Discussion

### Scientific production

Our results show a boom in research focused specifically on the oldest population starting in the mid-2000s, with a dramatic rise in the number of publications produced which has continued to the present day. A myriad of factors contributes to explaining the growth in scientific research output. First and foremost is the increased life expectancy and population aging [29]. At the same time, social and political consideration towards older people’s problems and conditions are favorable, with increasing investments in research projects focusing on concepts like healthy aging and well-being in the elderly [6, 8, 9]. Professional training in geriatrics has been developing, too, with the implementation of specialized education programs and the consolidation of this field as an established specialty, different from internal medicine and promoted by its own prestigious scientific societies [13].

The pronounced leadership of European research over that of the USA, whose output levels are below those seen in other biomedical research areas [30] is another notable finding from our analysis. At a country level, the importance of Japan, as the second biggest producer of research on very old populations, is noteworthy. As in Europe, this situation can be explained because Japan has one of the highest rates of population aging and among the longest life expectancies worldwide [31]. Logically then, researchers from these settings have typically occupied prominent positions in quantitative analyses of the scientific output on diseases like stroke, urinary tract infections, and eye diseases, which disproportionately affect older populations [20, 32, 33]. In contrast with other research areas, we did not observe a boom in publications from countries like China [34]. Our results, which point to a concentration of research in Europe and North America as well as in Japan, suggest the need to promote research focusing on very old populations in other settings, like southeast Asia, where the pace of population aging is accelerating [35].

### Research topic areas

The numerous disciplines linked to scientific output on very old populations confirm Mussi et al.’s [12] conclusions on the multidisciplinary nature of this research area. Indeed, the literature focusing on this collective is widely dispersed among specialized journals in different areas. Geriatrics and gerontology represent horizontal disciplines, treating elderly people who often have different, generalized problems associated with aging or geriatric syndromes. At the same time, old people also have diseases linked to other organs and organ systems that must be addressed by specialists from other disciplines, and this relationship is reflected in the corresponding research streams.

Dementia occupies a preeminent position in the research performed, as this is the main cause of dependence and disability among very old people [36]. In fact, in the UK the associated costs of this group of diseases exceed the combined costs incurred by heart diseases and cancer [37]. Since the turn of the 21st century, then, dementia has emerged as a priority for health systems in high-income countries [38].

Alzheimer’s disease is positioned as the main entity studied in research on age-associated dementias (*n* = 575), confirming the results reported by Serrano-Pozo et al. [17]. These authors analyzed the literature on Alzheimer’s disease published from 1975 to 2014, observing a steady growth of research output over the study period, especially in publications focused on the 80+ age group starting in the 1990s. Alzheimer’s disease is the most common type of dementia, accounting for up to 70% of all cases [6, 15, 39], and its prevalence and associated mortality has seen the most pronounced growth over the past several decades in the very old population [4]. Nevertheless, the relative dearth of research into other kinds of dementia (“Dementia, Vascular” *n* = 44; “Lewy Body Disease” *n* = 44; and negligible research in other pathologies) shows the need to promote research focusing on dementias other than Alzheimer’s in very old people. The scant research identified on “Frontotemporal dementia,” which is the second cause of dementia, is probably due to its earlier onset compared to Alzheimer’s [40].

Frailty constitutes another popular research topic associated with aging, as this is a very prevalent geriatric syndrome (≈ 30%) in people aged 80 years or older. Clinical manifestations include muscle weakness, fatigue, and slow motor performance, among others, and these are often accompanied by social vulnerability and dependence [6] along with a higher risk of falls, depression, disability, and mortality [41]. The ongoing COVID-19 pandemic stands out as the clearest example of how frailty can contribute decisively to situations of vulnerability in very old people and to a more severe clinical presentation of other diseases [42, 43].

The mingled impacts of different clinical and social factors underline why clinical research in very old people must necessarily take into account other social and technological considerations in order to reduce disease incidence and associated harms, both in patients and in their family members and caregivers [16]. Our findings on the relevance of caregiving, specialized care, ethics, and social and care support for patients with dementia and their family members corroborate those reported by Baldwin et al. [24].

More recently, Shi et al. [25] studied research on family caregivers of people with dementia, highlighting that the increased prevalence of dementia in very old people and their lengthening life expectancy can increase the risk of diverse health problems in family members, including depression, stress, anxiety, and other physical or psychological disorders. Caregivers have even been called “silent patients” or “secondary patients” whose needs should be addressed through specific strategies to protect and provide them with the required support; such strategies show unequal development across different countries [16]. For their part, Asghar et al. [44] studied assistive technology for people with dementia, signaling the relevance of telecare devices, activity monitoring, warning and reminder systems, fall detection, ease of mobility and communication, among other related aspects, tied to concepts like “independent living,” or “activities of daily living” in the present study.

The development of research in the area of fracture surgery is also notable, showing substantial growth over the past several years. Hip fractures are the most relevant surgical pathology in people of advanced age, due both to the high incidence and the potential for negative effects on quality of life, autonomy, and level of frailty in this collective. These impacts have driven important advances in areas like minimally invasive surgery and complex fracture management [19].

The descriptors referring to nutrition underscore the relevance of diet in relation with preventive and therapeutic treatments in very old people, as well as for addressing the deterioration of nutrition associated with factors like difficulty swallowing, loss of appetite, and reduced access to healthy diet [18]. Conversely, none of the clusters contain descriptors related with exercise or physical activity (e.g. “exercise” *n* = 121 or “exercise therapy” *n* = 57). Efforts to promote the integration of investigators from these areas into research on the pathologies identified could be warranted. Physical activity in relation to aging constitutes an area of active research, as Müller et al. [7] have pointed out. These authors report that the most highly cited literature in this area studies the association between physical activity and health-related outcomes, which may be a relevant factor for preventing dementia. Moreover, once the benefits of physical activity have been established, this study calls for continued research into designing effective and economically feasible interventions for physical activity. For their part, Gu et al. [6] draw attention to different factors in addition to physical activity, like emerging trends of research associated with healthy aging, such as diet, working memory, and active aging, pointing to the relevance that anti-aging medicine may have as a novel research field that considers all the above-mentioned aspects as well as genetic factors associated with aging and anti-aging pharmacological research.

Two prominent research clusters, focused on pathologies that are not specifically associated with very old populations, are devoted to cardiovascular diseases and neoplasms. Coronary artery disease and stroke, in particular, are the main causes of death worldwide and in all regions except Africa, explaining the importance of these areas in research [32].

Other cardiovascular diseases studied in very old patients are aneurysms and myocardial infarction, which follow stroke as the top cardiovascular diseases studied in this population. The rising prevalence of these diseases [45], along with major advances in research, are behind this trend, as described in different bibliometric studies [46–48]. The prevention and management of these pathologies also receive considerable attention [49], for instance the control of risk factors like hypertension [50] and the application of artificial intelligence as a decision-making tool for the clinical management of stroke and heart disease [51].

Likewise, a substantial body of research revolves around rehabilitation and patients’ adaptation to their new health state and living conditions following a cardiovascular event. Chow et al. [32] points out the importance of this research in driving improvements in patient outcomes. Indeed, the increase in scientific output on stroke contributed to reducing its associated mortality, and in turn, these positive trends have led researchers to direct more attention to aspects like pain management and rehabilitation.

The presence of lung and colorectal cancer in the cluster on neoplasms responds to the fact that these are among the cancers causing the highest mortality in very old people [3, 4]. On the other hand, the importance of skin neoplasms in our results is probably due to different factors, for example, the aggressiveness of melanomas among elderly people or the challenges for oncological practice derived from treatment complexity in people with multiple comorbidities [52]. Other important issues have to do with the fragility of aging skin and environmental factors like solar radiation, making skin care in very old people a priority for dermatological research [53].

The cluster on neoplasms also includes an important line of research on anti-vascular endothelial growth factor A (anti-VEGF) treatments, both for different cancers and for age-related macular degeneration. Although the latter is not a neoplasm, it is the primary cause of vision loss in old people and is mediated by VEGF. In any case, there are still many unanswered questions along this research stream, for example related to the best anti-VEGF medications or the identification of biomarkers that would enable better assessment of treatment efficacy [54].

Finally, the cluster around bacterial infections and anti-bacterial agents shows the importance of clinical entities like urinary tract infections and infections associated with the implantation of prostheses, especially those caused by staphylococci and *Klebsiella* spp., in very old people [55]. Urinary tract infections—often aggravated by bacterial resistance [56]—are the most common infections in this population due to the degenerative nature of the urinary tract [57], and they are also responsible for worsening patients’ functional status. Research addressing this problem has seen notable growth in the past several years around questions like multidrug resistance and the most appropriate treatments for different types of infections [33].

### Limitations

The main limitation of this analysis is that quantitative studies like ours provide a vision based on the research generating the most publications; however, they do not necessarily capture prominent lines of research on emerging or highly specific pathologies. Our results may also underestimate the body of research on diseases that generally develop before people reach such an advanced age, like diabetes, osteoporosis, chronic obstructive pulmonary disease, or Parkinson’s disease. The individual descriptors for these diseases all stand out as important, but they are not integrated in any of the thematic clusters we identified. Other highly prevalent conditions, for example eye diseases like glaucoma or cataracts, are underrepresented in our findings because there are already well-established pharmacological or surgical treatments available for them, so they attract less research interest. Our use of a descriptor for a human age group led to the exclusion of a large volume of literature that does not exclusively focus on the oldest age groups and probably most basic research that is not explicitly linked to studying very advanced age as a key variable. Finally, our analysis may not have picked up on the full body of research in some psychosocial areas, as it was based on terminology from the MeSH thesaurus, which is mainly rooted in biomedical fields.

In addition, although we observed some very well-defined thematic clusters, multidisciplinary approaches to different pathologies remain vitally important in this population. Not only do a large proportion of very old people have multiple comorbidities, geriatric syndromes like sarcopenia or other conditions provoking the loss of skeletal mass can act as risk factors for pathologies like cardiovascular diseases. These associations are sometimes underrepresented in research [18].

## Conclusions

Despite the above limitations, this study contributes to advancing knowledge on the research performed in the context of population aging, helping to remedy the near-total absence of studies offering a comprehensive vision of the research panorama in this field. The main conclusions about the research related to the oldest population groups (≥ 80 years) are as follows:

- The pace of scientific output began to speed up in the mid 2000s, although research is highly concentrated in the USA, Japan, and some European populations—settings where the greatest proportion of very old people live. Given that the highest rates of population aging in the coming decades will be in low- and middle-income countries, more balanced participation in this research should be promoted among different regions and countries.

- Our results demonstrate the complexity of research in this area, which brings together numerous medical specialties and studies on different pathologies. In quantitative terms, the most prominent clusters focus on cardiovascular and cerebrovascular diseases, such as aortic valve stenosis and stroke; dementia— particularly Alzheimer’s disease; neoplasms with a higher incidence in the oldest populations; and bacterial infections in the urinary tract and prostheses. However, we also identified a number of other important clusters, focusing on a myriad of diseases, degenerative pathologies, and geriatric syndromes that need to be addressed in conjunction with social and psychosocial aspects related to aging.

## Supplementary Information

**Additional file 1.** Main terms referring to diseases linked with the MeSH descriptor “Aged, 80 and over”. Description of data: List of MeSH terms for diseases assigned to at least 50 of the documents on people aged 80 and over, with indicators for overall output and the number of documents that focus on the topic specifically for this age group.

## Data Availability

The datasets used and/or analysed during the current study are available from the corresponding author on reasonable request.
