# Supplementary material for: Thematic research clusters in very old populations (≥ 80 years): a bibliometric approach
Source: BMC Geriatr. 2021 Apr 21;21:266. doi: 10.1186/s12877-021-02209-7 (PMC8058755; doi:10.1186/s12877-021-02209-7)
Supplement: Supplementary file 1 — Additional file 1. Main terms referring to diseases linked with the MeSH descriptor “Aged, 80 and over”. Description of data: List of MeSH terms for diseases assigned to at least 50 of the documents on people aged 80 and over, with indicators for overall output and the number of documents that focus on the topic specifically for this age group. [file 12877_2021_2209_MOESM1_ESM.docx]

**TITLE PAGE (ADDITIONAL FILE 1)**

**Thematic research clusters in very old populations (≥ 80 years): a bibliometric approach**

Authors: Gregorio Gonzalez-Alcaide^a^†, Sergio Palacios-Fernandez^b^†, Jose-Manuel Ramos-Rincon^c,d^

1. Department of History of Science and Documentation, University of Valencia. Valencia, Spain.
2. Department of Internal Medicine, Hospital Universitari Sant Joan d’Alacant. Alicante, Spain
3. Department of Internal Medicine, General University Hospital of Alicante-ISABIAL. Alicante, Spain.
4. Department of Clinical Medicine, Miguel Hernandez University of Elche. Alicante, Spain.

† Equal contributor

Corresponding author: Gregorio González Alcaide (gregorio.gonzalez@uv.es)

**Additional file 1. Main terms referring to diseases linked with the MeSH descriptor “Aged, 80 and over”**

| **MeSH descriptor** | **Aged, 80 and over**  **(exclusively age group)** | | **Aged, 80 and over** | |
| --- | --- | --- | --- | --- |
|  | **N** | **%** | **N** | **%** |
| Aortic Valve Stenosis | 1134 | 5,14 | 5594 | 0,79 |
| Skin Neoplasms | 951 | 4,31 | 9993 | 1,41 |
| Dementia | 697 | 3,16 | 11572 | 1,64 |
| Alzheimer Disease | 575 | 2,61 | 16802 | 2,38 |
| Stroke | 500 | 2,27 | 19946 | 2,82 |
| Carcinoma, Squamous Cell | 435 | 1,97 | 15093 | 2,13 |
| Adenocarcinoma | 417 | 1,89 | 16172 | 2,29 |
| Accidental Falls | 376 | 1,71 | 6680 | 0,94 |
| Lung Neoplasms | 365 | 1,66 | 19306 | 2,73 |
| Cognition Disorders | 365 | 1,66 | 9560 | 1,35 |
| Heart Failure | 352 | 1,60 | 9650 | 1,36 |
| Hypertension | 325 | 1,47 | 10112 | 1,43 |
| Hip Fractures | 323 | 1,47 | 5759 | 0,81 |
| Myocardial Infarction | 295 | 1,34 | 8284 | 1,17 |
| Aortic Aneurysm, Abdominal | 270 | 1,22 | 4153 | 0,59 |
| Prosthesis Failure | 264 | 1,20 | 4789 | 0,68 |
| Melanoma | 253 | 1,15 | 6395 | 0,90 |
| Hemorrhage | 221 | 1,00 | 3801 | 0,54 |
| Depression | 218 | 0,99 | 10078 | 1,43 |
| Coronary Artery Disease | 215 | 0,98 | 5678 | 0,80 |
| Cardiovascular Diseases | 199 | 0,90 | 9578 | 1,35 |
| Breast Neoplasms | 198 | 0,90 | 20076 | 2,84 |
| Carcinoma, Basal Cell | 197 | 0,89 | 2211 | 0,31 |
| Stomach Neoplasms | 195 | 0,88 | 8992 | 1,27 |
| Neoplasm Recurrence, Local | 192 | 0,87 | 17501 | 2,47 |
| Prostatic Neoplasms | 168 | 0,76 | 11238 | 1,59 |
| Liver Neoplasms | 167 | 0,76 | 10517 | 1,49 |
| Cognitive Dysfunction | 163 | 0,74 | 5305 | 0,75 |
| Calcinosis | 163 | 0,74 | 2100 | 0,30 |
| Neoplasm Invasiveness | 162 | 0,73 | 9715 | 1,37 |
| Thrombosis | 161 | 0,73 | 2374 | 0,34 |
| Gastrointestinal Hemorrhage | 158 | 0,72 | 2727 | 0,39 |
| Colonic Neoplasms | 156 | 0,71 | 3948 | 0,56 |
| Hematoma | 155 | 0,70 | 1334 | 0,19 |
| Aneurysm, False | 153 | 0,69 | 758 | 0,11 |
| Neoplasms, Multiple Primary | 153 | 0,69 | 1395 | 0,20 |
| Lymphoma, Large B-Cell, Diffuse | 150 | 0,68 | 2303 | 0,33 |
| Diabetes Mellitus, Type 2 | 150 | 0,68 | 7944 | 1,12 |
| Aortic Valve Insufficiency | 148 | 0,67 | 1037 | 0,15 |
| Delirium | 147 | 0,67 | 1873 | 0,26 |
| Bacteremia | 144 | 0,65 | 2757 | 0,39 |
| Kidney Failure, Chronic | 144 | 0,65 | 5193 | 0,73 |
| Mitral Valve Insufficiency | 143 | 0,65 | 1354 | 0,19 |
| Acute Kidney Injury | 143 | 0,65 | 2142 | 0,30 |
| Pancreatic Neoplasms | 139 | 0,63 | 6734 | 0,95 |
| Head and Neck Neoplasms | 132 | 0,60 | 6085 | 0,86 |
| Staphylococcal Infections | 132 | 0,60 | 2694 | 0,38 |
| Aortic Aneurysm, Thoracic | 130 | 0,59 | 1730 | 0,24 |
| Dyspnea | 130 | 0,59 | 1716 | 0,24 |
| Brain Ischemia | 130 | 0,59 | 5965 | 0,84 |
| Macular Degeneration | 126 | 0,57 | 3567 | 0,50 |
| Lymphatic Metastasis | 126 | 0,57 | 13505 | 1,91 |
| Intestinal Obstruction | 126 | 0,57 | 1577 | 0,22 |
| Deglutition Disorders | 124 | 0,56 | 2467 | 0,35 |
| Urinary Bladder Neoplasms | 123 | 0,56 | 5565 | 0,79 |
| Ischemia | 123 | 0,56 | 2710 | 0,38 |
| Osteoporosis | 122 | 0,55 | 4669 | 0,66 |
| Abdominal Pain | 120 | 0,54 | 1181 | 0,17 |
| Takotsubo Cardiomyopathy | 119 | 0,54 | 473 | 0,07 |
| Carcinoma | 117 | 0,53 | 5860 | 0,83 |
| Inflammation | 113 | 0,51 | 3824 | 0,54 |
| Rare Diseases | 113 | 0,51 | 406 | 0,06 |
| Malnutrition | 113 | 0,51 | 1957 | 0,28 |
| Fractures, Bone | 113 | 0,51 | 3660 | 0,52 |
| Pulmonary Embolism | 112 | 0,51 | 2814 | 0,40 |
| Heart Diseases | 111 | 0,50 | 2446 | 0,35 |
| Spinal Fractures | 110 | 0,50 | 2853 | 0,40 |
| Prosthesis-Related Infections | 110 | 0,50 | 2295 | 0,32 |
| Necrosis | 109 | 0,49 | 1372 | 0,19 |
| Kidney Neoplasms | 108 | 0,49 | 5405 | 0,76 |
| Colorectal Neoplasms | 105 | 0,48 | 13645 | 1,93 |
| Iatrogenic Disease | 105 | 0,48 | 885 | 0,13 |
| Parkinson Disease | 104 | 0,47 | 6440 | 0,91 |
| Urinary Tract Infections | 103 | 0,47 | 1946 | 0,28 |
| Bone Neoplasms | 101 | 0,46 | 3145 | 0,44 |
| Colectomy | 101 | 0,46 | 2674 | 0,38 |
| Pulmonary Disease, Chronic Obstructive | 98 | 0,44 | 5354 | 0,76 |
| Acute Coronary Syndrome | 97 | 0,44 | 1949 | 0,28 |
| Aortic Rupture | 95 | 0,43 | 1154 | 0,16 |
| Femoral Neck Fractures | 94 | 0,43 | 1477 | 0,21 |
| Neoplasms | 93 | 0,42 | 13360 | 1,89 |
| Gallstones | 92 | 0,42 | 1127 | 0,16 |
| Pneumonia | 91 | 0,41 | 2662 | 0,38 |
| Neoplasm Metastasis | 91 | 0,41 | 7220 | 1,02 |
| Carotid Stenosis | 91 | 0,41 | 2996 | 0,42 |
| Sepsis | 91 | 0,41 | 2755 | 0,39 |
| Renal Insufficiency, Chronic | 90 | 0,41 | 3042 | 0,43 |
| Coronary Stenosis | 88 | 0,40 | 1128 | 0,16 |
| Aneurysm, Ruptured | 88 | 0,40 | 994 | 0,14 |
| Giant Cell Arteritis | 87 | 0,39 | 789 | 0,11 |
| Neoplasms, Second Primary | 85 | 0,39 | 1683 | 0,24 |
| Diabetes Mellitus | 85 | 0,39 | 6030 | 0,85 |
| Thyroid Neoplasms | 84 | 0,38 | 3355 | 0,47 |
| Intestinal Perforation | 84 | 0,38 | 870 | 0,12 |
| Aortic Aneurysm | 84 | 0,38 | 1161 | 0,16 |
| Rupture, Spontaneous | 84 | 0,38 | 595 | 0,08 |
| Heart Valve Diseases | 83 | 0,38 | 1406 | 0,20 |
| Leukemia, Lymphocytic, Chronic, B-Cell | 81 | 0,37 | 2344 | 0,33 |
| Carcinoma, Merkel Cell | 81 | 0,37 | 724 | 0,10 |
| Carcinoma, Non-Small-Cell Lung | 80 | 0,36 | 9046 | 1,28 |
| Sarcopenia | 79 | 0,36 | 1360 | 0,19 |
| Femoral Fractures | 79 | 0,36 | 1504 | 0,21 |
| Anemia | 78 | 0,35 | 2264 | 0,32 |
| Kidney Diseases | 78 | 0,35 | 2573 | 0,36 |
| Carcinoma, Renal Cell | 77 | 0,35 | 4022 | 0,57 |
| Cerebral Infarction | 76 | 0,34 | 1645 | 0,23 |
| Pleural Effusion | 76 | 0,34 | 938 | 0,13 |
| Endocarditis, Bacterial | 75 | 0,34 | 646 | 0,09 |
| Constriction, Pathologic | 75 | 0,34 | 1733 | 0,25 |
| Multiple Myeloma | 75 | 0,34 | 2916 | 0,41 |
| Hemangiosarcoma | 75 | 0,34 | 424 | 0,06 |
| Vision Disorders | 74 | 0,34 | 2143 | 0,30 |
| Esophageal Neoplasms | 74 | 0,34 | 4974 | 0,70 |
| Rectal Neoplasms | 74 | 0,34 | 4583 | 0,65 |
| Drug Eruptions | 74 | 0,34 | 526 | 0,07 |
| Eyelid Neoplasms | 74 | 0,34 | 560 | 0,08 |
| Carcinoma, Hepatocellular | 74 | 0,34 | 5876 | 0,83 |
| Arthritis, Rheumatoid | 73 | 0,33 | 3787 | 0,54 |
| Aortic Diseases | 72 | 0,33 | 1123 | 0,16 |
| Depressive Disorder | 72 | 0,33 | 3547 | 0,50 |
| Gram-Positive Bacterial Infections | 72 | 0,33 | 936 | 0,13 |
| Abscess | 72 | 0,33 | 558 | 0,08 |
| Hallucinations | 72 | 0,33 | 533 | 0,08 |
| Critical Illness | 72 | 0,33 | 3120 | 0,44 |
| Aneurysm | 70 | 0,32 | 637 | 0,09 |
| Mobility Limitation | 69 | 0,31 | 1765 | 0,25 |
| Amyloidosis | 69 | 0,31 | 723 | 0,10 |
| Endoleak | 69 | 0,31 | 666 | 0,09 |
| Surgical Wound Infection | 68 | 0,31 | 2891 | 0,41 |
| Thrombocytopenia | 68 | 0,31 | 1150 | 0,16 |
| Pneumonectomy | 67 | 0,30 | 2026 | 0,29 |
| Skin Diseases | 67 | 0,30 | 1143 | 0,16 |
| Disease Management | 67 | 0,30 | 1962 | 0,28 |
| Heart Arrest | 66 | 0,30 | 1194 | 0,17 |
| Pressure Ulcer | 65 | 0,29 | 1336 | 0,19 |
| Cross Infection | 65 | 0,29 | 3845 | 0,54 |
| Venous Thrombosis | 65 | 0,29 | 2657 | 0,38 |
| Facial Neoplasms | 65 | 0,29 | 535 | 0,08 |
| Atrioventricular Block | 65 | 0,29 | 345 | 0,05 |
| Klebsiella Infections | 63 | 0,29 | 642 | 0,09 |
| Nose Neoplasms | 63 | 0,29 | 772 | 0,11 |
| Hypotension | 63 | 0,29 | 841 | 0,12 |
| Vulvar Neoplasms | 62 | 0,28 | 808 | 0,11 |
| Sweat Gland Neoplasms | 62 | 0,28 | 235 | 0,03 |
| Pancreatitis | 60 | 0,27 | 1590 | 0,22 |
| Arrhythmias, Cardiac | 60 | 0,27 | 1465 | 0,21 |
| Cerebral Hemorrhage | 60 | 0,27 | 2446 | 0,35 |
| Intestinal Fistula | 60 | 0,27 | 448 | 0,06 |
| Lung Diseases | 59 | 0,27 | 1595 | 0,23 |
| Brain Neoplasms | 59 | 0,27 | 4666 | 0,66 |
| Tachycardia, Ventricular | 59 | 0,27 | 589 | 0,08 |
| Airway Obstruction | 59 | 0,27 | 528 | 0,07 |
| Diabetes Complications | 59 | 0,27 | 2720 | 0,38 |
| Coronary Disease | 58 | 0,26 | 3122 | 0,44 |
| Hernia, Inguinal | 57 | 0,26 | 1074 | 0,15 |
| Hypoglycemia | 57 | 0,26 | 795 | 0,11 |
| Mental Disorders | 57 | 0,26 | 3830 | 0,54 |
| Psychomotor Agitation | 56 | 0,25 | 645 | 0,09 |
| Stress, Psychological | 56 | 0,25 | 4729 | 0,67 |
| Peritonitis | 56 | 0,25 | 783 | 0,11 |
| Ovarian Neoplasms | 56 | 0,25 | 4690 | 0,66 |
| Heart Neoplasms | 56 | 0,25 | 388 | 0,05 |
| Wet Macular Degeneration | 55 | 0,25 | 975 | 0,14 |
| Gram-Negative Bacterial Infections | 55 | 0,25 | 849 | 0,12 |
| Coronary Occlusion | 55 | 0,25 | 247 | 0,03 |
| Soft Tissue Neoplasms | 55 | 0,25 | 1203 | 0,17 |
| Edema | 54 | 0,24 | 818 | 0,12 |
| Adenoma | 54 | 0,24 | 2943 | 0,42 |
| Parotid Neoplasms | 54 | 0,24 | 702 | 0,10 |
| Escherichia coli Infections | 54 | 0,24 | 933 | 0,13 |
| Streptococcal Infections | 53 | 0,24 | 751 | 0,11 |
| Myocardial Ischemia | 53 | 0,24 | 2005 | 0,28 |
| Carcinoma, Transitional Cell | 53 | 0,24 | 2462 | 0,35 |
| Intraocular Pressure | 53 | 0,24 | 4048 | 0,57 |
| Sarcoma | 53 | 0,24 | 1654 | 0,23 |
| Corneal Diseases | 53 | 0,24 | 1100 | 0,16 |
| Postoperative Hemorrhage | 53 | 0,24 | 1446 | 0,20 |
| Iliac Aneurysm | 52 | 0,24 | 333 | 0,05 |
| Carcinoma, Papillary | 52 | 0,24 | 2008 | 0,28 |
| Atrophy | 52 | 0,24 | 1906 | 0,27 |
| Arthritis, Infectious | 52 | 0,24 | 482 | 0,07 |
| Skin Ulcer | 52 | 0,24 | 465 | 0,07 |
| Obesity | 52 | 0,24 | 6171 | 0,87 |
| Hyponatremia | 52 | 0,24 | 576 | 0,08 |
| Chest Pain | 51 | 0,23 | 870 | 0,12 |
| Embolism | 50 | 0,23 | 635 | 0,09 |
| Syncope | 50 | 0,23 | 630 | 0,09 |
| Gastrointestinal Stromal Tumors | 50 | 0,23 | 1147 | 0,16 |
